# Higher Body Fat and Lower Fat-Free Mass in Girls with Premature Adrenarche

**DOI:** 10.4274/jcrpe.1525

**Published:** 2015-03-05

**Authors:** Ayşe Nurcan Cebeci, Ayşegül Taş

**Affiliations:** 1 Derince Training and Research Hospital, Clinic of Pediatric Endocrinology, Kocaeli, Turkey; 2 Derince Training and Research Hospital, Clinic of Physiology, Kocaeli, Turkey

**Keywords:** Premature adrenarche, Pubarche, body fat, bioelectrical impedance, puberty, fat-free mass

## Abstract

**Objective:**

Idiopathic premature adrenarche (PA) refers to presence of androgenic signs before the age of eight years in girls in the absence of thelarche. In children with PA, increased adrenal androgens lead to changes in body composition and transient growth acceleration. Although the association between PA and some components of the metabolic syndrome is well known, body composition has not been extensively studied in these patients.

**Methods:**

We examined 47 girls with PA with a median age of 7.39 years and 57 healthy controls with a median age of 7.11 years. For PA group, the inclusion criteria were appearance of pubic/axillary hair before 8 years of age, absence of findings of central puberty and absence of use of any medication. Patients with steroidogenic enzyme defects and virilizing tumors were excluded. Height, body weight, waist and hip circumference were measured. The bioelectrical impedance method was used for body composition analysis.

**Results:**

In the PA group, both body weight standard deviation score (SDS) and height SDS were significantly higher than in the controls (p<0.001 for both). While total body fat percentage values were significantly higher in the PA group than in the controls (median 22.8% vs. 19.95%, p=0.049), fat-free mass (FFM) and total muscle mass percentages were significantly lower than in the controls (median 76.8% vs. 79.9%, p=0.024 and 72.6% vs. 75.7%, p=0.018, respectively).

**Conclusion:**

Our findings revealed that girls with PA have higher body weight and height for age values. They also show significant changes in body composition such as an increase in total body fat percentage with a concomitant decrease in the percentages of FFM, muscle mass and total body water.

## INTRODUCTION

Premature adrenarche (PA) refers to the development of pubic/axillary hair before the age of eight years in girls in the absence of thelarche. PA is often accompanied by adult apocrine body odor, oily hair and skin (1). Although PA originally has been considered a benign condition (2), increasing body of evidence suggests that PA may be a “forerunner of the metabolic syndrome” (3). Utriainen et al (4) demonstrated that Finnish girls with PA have excessive weight and show an increased prevalence of childhood metabolic syndrome. In 1998, Ibáñez et al (5) first stated the association of PA with hyperinsulinism and ovarian hyperandrogenism. This occurs more prominently in girls with reduced fetal growth followed by rapid weight gain (6,7). It has been suggested that girls with PA who remain obese are at higher risk for developing polycystic ovary syndrome (8,9,10).

On the other hand, even in children with normal birth weight, being obese at seven years of age is found to be associated with increased adrenal androgen levels (11). Moreover, obese children with higher adrenal androgens are reported to be fatter and more centrally obese than their counterparts (11).

Increased adrenal androgens may lead to changes in body composition and transient growth acceleration in girls with PA (1). To the best of our knowledge, there is only one study in which the changes in body composition in girls with PA have been investigated (12). This study showed that these girls had excess total body and central fat mass (FM) throughout all pubertal stages. PA has been suggested to be a feature of the metabolic complications of obesity (13).

In the present study, our aim was to determine whether any significant changes in body composition occur in Turkish girls with PA.

## METHODS

Children who were referred to the Pediatric Endocrinology Clinic of Kocaeli Derince Training and Research Hospital for evaluation of adrenarche between the dates of November 2013 and April 2014 were consecutively assessed. For the subjects with PA, the inclusion criteria were the appearance of pubic/axillary hair before age 8 years, the absence of thelarche and absence of any medication use. Steroidogenic enzyme defects and virilizing tumors were excluded by hormonal studies and by adrenal ultrasonography. Healthy volunteers of first-second grade at an elementary school from the same district constituted the control group. The criteria for entry into the control group included absence of pubic/axillary hair and of thelarche on physical examination and no history of any chronic disease or medication. There were 47 subjects aged between 6.22 and 8.30 years in the PA group and 57 subjects aged between 6.31 and 8.25 years in the control group. All patients and controls were informed about the study and written informed consent was obtained from the parents. The study was approved by the Ethics Committee of the School of Medicine, Kocaeli University, Kocaeli, Turkey (Reference number: KOU KAEK 2013/16).

All patients and controls were examined by the same pediatric endocrinologist (ANC); anthropometric measurements and pubertal stages were recorded. Height and weight measurements of the study group were performed with the subject in light clothes and wearing no shoes, using a combined stadiometer and calibrated electronic scale with 0.1 cm and 0.1 kg sensitivity (Tess EBB, Comak Tartı, Turkey). Body mass index (BMI) was calculated by using the formula of body weight (kg) / height2 (m2). The Turkish references for height, weight and BMI were used in the calculations of standard deviation scores (SDS) (14). Obesity was defined as BMI SDS over two.

Waist circumference (WC) was measured around the smallest area of the waist, approximately one inch (2.54 cm) above the umbilicus. Hip circumference (HC) measurement was taken around the largest area of the buttocks. An elastic measuring tape was used to measure WC and HC. A WC value ≥90th for age and gender, using the national data for WC, was taken as a measure of central obesity (15).

### Body Composition Analysis

We used the Tanita (model MC-780MA; Tanita, Tokyo, Japan) bioimpedance segmental body composition analyzer to assess body composition in all subjects. This device calculates total body weight, BMI, body fat percentage (%), total body FM and muscle mass (kg), total body water, truncal (core) FM and muscle mass on the basis of data using bioelectrical impedance analysis (BIA) through the use of 8 electrodes (8-contacts; two on each hand and foot). We applied the measurement procedure as described in previous studies (16,17,18).

### Data Analysis

The data were analyzed using the SPSS version 20 statistical package program (SPSS, Chicago, IL, USA). Kolmogorov-Smirnov test was used to test the normality of the data. All data were expressed as median (Interquartile range-IQR) values. The Mann-Whitney U test was used for comparisons and Spearman’s correlation method was used in correlation analysis. Comparison of categorical variables was performed using Pearson’s chi-square test. For all tests, significance was evaluated at p<0.05.

## RESULTS

The characteristics of the groups are demonstrated in Table 1. There were no differences regarding age and BMI or BMI SDS between the two groups (p>0.05 for all). Girls with PA had higher height and body weight values as well as higher height and weight SDSs than the control group (p<0.001 for both). In the PA group, 10 girls (21.3%) were obese, whereas obesity was found in only 2 girls (3.5%) in the control group. Central obesity was found in 18 subjects (38.3%) in the PA group and in 11 controls (19.3%) (p=0.032).

Although both WC and HC values were higher in girls with PA, waist-to-hip ratio was found to be higher in the control group (waist-to-hip ratio: 0.86 in PA group and 0.89 in controls, p=0.018).

Body composition analysis results are shown in Table 2. In the PA group, FM, muscle mass, total body water and free FM (FFM) were all found to be significantly higher than the controls. However, body fat percentage in the PA group was only a slightly higher than that of the controls (median 22.8% vs. 19.95%, p=0.049). The PA group had a significantly lower muscle mass and FFM percentage than controls (median 72.6% vs. 75.7%, p=0.018 and median 76.8% vs. 79.9%, p=0.024, respectively). The percentage of total body water in the PA group was also significantly less than the controls (56.4% vs. 58.6%, p=0.018). WC was positively correlated with truncal fat (r=0.865, p<0.001) and FFM (r=0.828, p<0.001) in the whole group.

## DISCUSSION

The present study shows increased body fat and decreased FFM in girls with idiopathic PA. In accordance with previous findings (19,20,21), there was a significant acceleration in growth in our study group at the time of diagnosis. There was also a modest increase in skeletal age (data not shown); however, this finding was consistent with height age. Both height and body weight of girls with PA were significantly increased, while BMI values were comparable in the two groups . This finding was similar to the findings of Ibáñez et al (12) which showed that the BMI values of girls with premature pubarche aged between 6-18 years were not any different from those of control girls matched for age and pubertal stage. These authors also reported that girls with premature pubarche had higher total FM, percentage FM, abdominal FM and truncal FM than the controls at each pubertal stage. FFM was not evaluated in this study.

Our girls with PA had slightly higher body fat percentage values than the controls. However, the decrease in FFM was more distinctive. In our study, muscle mass percentage and total body water percentage were also found to be decreased in PA girls. It is important to note that body composition analyses were performed at the time of diagnosis in all children and the median age at diagnosis was 7.39 years. The changes in body composition occur very early in PA, therefore we suggest that these girls should be monitored for metabolic complications, ovarian hyperandrogenism and ovulatory dysfunction.

It is known that central or visceral fat is associated with hyperinsulinemia and future cardiovascular diseases (22,23). For this reason, it is important to determine the fat distribution in girls with PA. Women with hyperandrogenemia and polycyctic ovary syndrome have central or “android” fat distribution and the same type of fat distribution has been shown in girls with a history of premature pubarche (12,24). Our subjects with PA had a significantly higher percentage of central obesity than control subjects. Yet, in contrast to findings of previous reports (12), the waist-to-hip ratio was not higher in girls with PA in our study. Since an increase in both total fat and truncal fat were found in our PA group, one might consider that the increased fat accumulated more in the hip area and inner thighs as seen in a so-called “gynecoid” fat distribution. The pattern of fat distribution might be related to ethnic differences and feeding style, but this issue remains to be clarified in future studies (25,26).

The prevalence of obesity was much higher among our PA girls. Previous studies demonstrated an association between increasing adiposity and elevated adrenal androgens during childhood (20,27). l’Allemand et al (27) concluded that neither BMI nor leptin levels can explain the increased androgen secretion in children with PA. Hyperinsulinism due to insulin resistance may play a role, yet no correlation between insulin and dehydroepiandrosterone sulfate levels was found in prepubertal children (28). The prevalence of obesity (defined as a BMI SDS ≥ 2) has been reported as 4.1% among seven years old Turkish girls (29). Our study group had a higher prevalence than this reported value, while our control group had a much lower prevalence. Our findings confirm the link between obesity and PA in Turkish girls.

The strengths of our study include the investigation of body composition of Turkish girls for the first time. Another strong point of the study is having an age-matched control group. We did not exclude underweight or overweight children from the controls; hence our control group was not matched for weight. In the study design, we thought that a community-based random sample group of healthy children could allow us to make a better comparison regarding prevalence of obesity associated with PA.

Our study also had some limitations. First, body composition and fat distribution were assessed with BIA and not with the “gold-standard” method dual-energy X-ray absorptiometry (DEXA). We have chosen BIA for our study since it is a simple, safe and inexpensive method for assessment of body composition (30). This method has been validated against gold-standard DEXA in pediatric settings (16,30). Another limitation of our study is its cross-sectional design, which did not allow us to establish a causal link. Last but not least, we were not able to take a birth weight and early weight gain history from all subjects and therefore we could not include these data to the analyses.

To conclude, Turkish girls with idiopathic PA showed an increase in body weight and an accelerated height growth even at diagnosis. They also demonstrated significant changes in body composition such as an increase in total body fat percentage with a concomitant decrease in the percentages of FFM, muscle mass and total body water.

## Figures and Tables

**Table 1:**
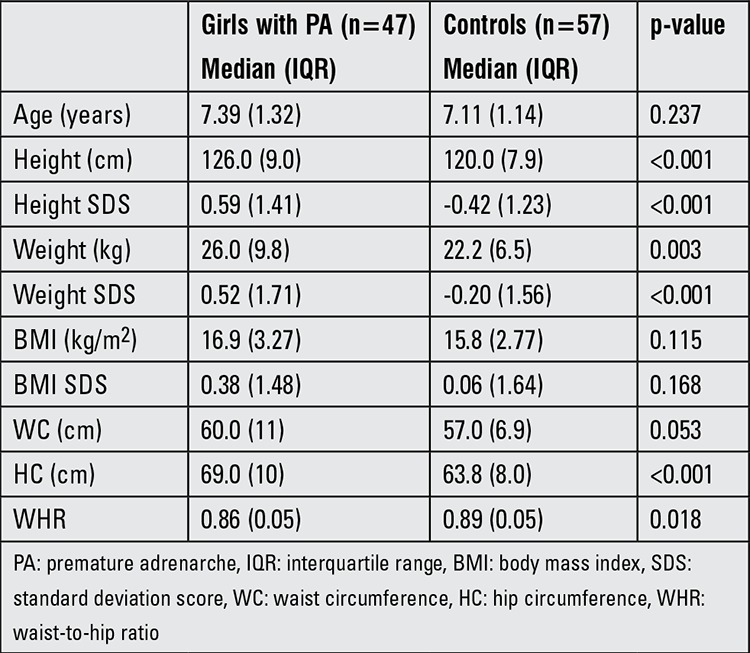
Age and anthropometric measurements of the study group

**Table 2:**
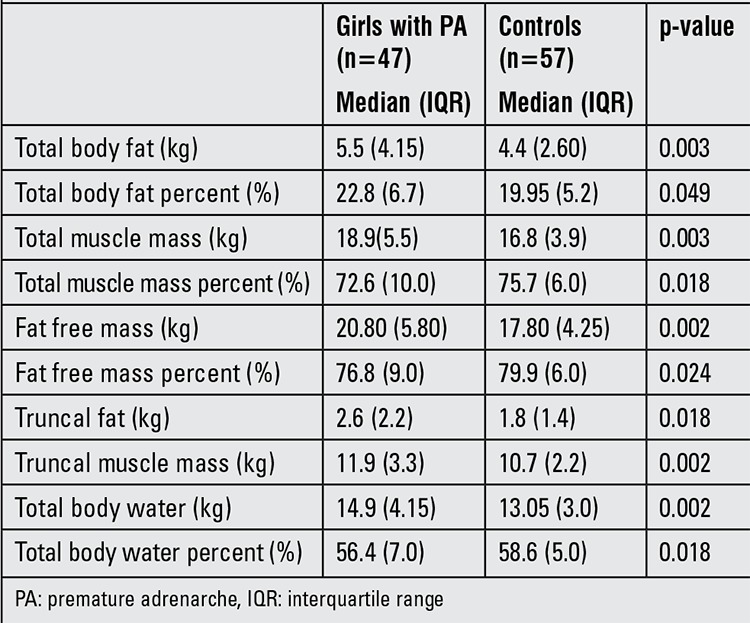
Bioelectric impedance analysis values of the study and control groups
